# Parasite infection accelerates age polyethism in young honey bees

**DOI:** 10.1038/srep22042

**Published:** 2016-02-25

**Authors:** Antoine Lecocq, Annette Bruun Jensen, Per Kryger, James C. Nieh

**Affiliations:** 1 Division of Biological Sciences, Section of Ecology, Behavior, and Evolution, University of California San Diego, 9500 Gilman Drive, MC 0116, La Jolla, California, 92093–0166, United States of America; 2 University of Copenhagen, Department of Plants and Environmental Sciences - PLEN, Thorvaldsensvej 40, 1871 Frederiksberg C, DK; 3 Aarhus University, Department of Agroecology - Entomology and Plant Pathology, Forsøgsvej 1, 4200 Slagelse, DK.

## Abstract

Honey bees (*Apis mellifera*) are important pollinators and their health is threatened worldwide by persistent exposure to a wide range of factors including pesticides, poor nutrition, and pathogens. *Nosema ceranae* is a ubiquitous microsporidian associated with high colony mortality. We used lab micro-colonies of honey bees and video analyses to track the effects of *N. ceranae* infection and exposure on a range of individual and social behaviours in young adult bees. We provide detailed data showing that *N. ceranae* infection significantly accelerated the age polyethism of young bees, causing them to exhibit behaviours typical of older bees. Bees with high *N. ceranae* spore counts had significantly increased walking rates and decreased attraction to queen mandibular pheromone. Infected bees also exhibited higher rates of trophallaxis (food exchange), potentially reflecting parasite manipulation to increase colony infection. However, reduction in queen contacts could help bees limit the spread of infection. Such accelerated age polyethism may provide a form of behavioural immunity, particularly if it is elicited by a wide variety of pathogens.

The Western honey bee, *Apis mellifera*, is a key global pollinator of cultivated and native plants, but the managed bee population has experienced concerning declines^1^. A variety of pathogens and parasites, xenobiotics and other environmental stressors leading to nutritional deficiencies, and their interactions contribute to high annual colony losses^1^. The microsporidian parasite *Nosema ceranae* has drawn particular attention, in part because it is so widespread^2^,^3^,^4^ and is often associated with colony losses^5^,^6^. Although its exact role in recent colony losses has been debated^3^,^6^, *N. ceranae* is now one of the most globally prevalent honey bee pathogens. *Nosema sp.* primarily contributes to poor colony health, rendering colonies more susceptible to other stressors^7^. In combination with other factors, such as pesticides, *Nosema* infection can reduce colony survival^8^,^9^.

*Nosema ceranae* is a spore forming fungus that lives as an obligate intracellular pathogen and exists outside the host cell as metabolically inactive spores^10^. It infects and reproduces inside epithelial cells of the midgut, but has also been showed to infect other tissues^11^,^12^. Spores are produced and released into the environment when an infected bee defecates. These spores are a source of infection for other bees, following a typical faecal-oral transmission pathway^13^. However *N. ceranae* may also be spread orally, through trophallaxis^14^, be transmitted through cleaning and grooming^4^, and sexually^15^.

*Nosema ceranae* infection can reduce bee longevity and leads to gut tissue degeneration^6^ and nutritional stress^16^. However, *Nosema* infection can also alter age polyethism, the natural progression of tasks that honey bees perform as they grow older. During the first 12–15 days of adult life, workers typically carry out nest activities such as cleaning, brood and queen tending, comb building and food handling. Ventilation and guard duty behaviours peak at around 18 days of age and the final task, foraging, typically peaks at day 23^17^. Bees infected with *Nosema* tend to forage precociously^13^,^18^,^19^ and a preliminary study suggested that other activities typically performed by very young bees are similarly time-shifted forward^20^. Recently, Natsopoulou *et al.*^21^ demonstrated that starting at 4 days of age, *N. ceranae* accelerated the temporal polyethism schedule, that infected bees displayed increased hyperactivity and foraging-related tasks but without reducing host behavioural repertoire. However, it is unclear how *Nosema* infection alters the normal progression of age polyethism in young bees.

An intriguing question is whether behavioural shifts caused by *Nosema sp.* infection benefit the parasite or the host. The parasite may manipulate host behaviour. Infected bees gravitate towards the higher temperatures of the inner hive, an area of high bee density and could thereby enhance parasite spread^22^. However, the host has defences. In addition to their innate immunity, honey bees exhibit behavioural immunity^23^ through actions such as hygienic removal of diseased bees^24^ and allogrooming^25^. Other behaviours that limit the spread of disease, such as precocious foraging (a form of self-removal^26^) could also reduce disease spread inside a colony. Finally, behaviours that limit exposure of the queen should protect the colony. The queen is an interaction centre and focal point of colony life. She is primarily attended by young adults that are particularly drawn to her queen mandibular pheromone (QMP)^27^. Do bees infected with *Nosema* attempt to enhance colony behavioural immunity or reduce disease spread?

We therefore tested the hypothesis that *Nosema* infection will accelerate the age polyethism of young bees and decrease contact between infected workers and the queen. To study age polyethism, we used a group of bees of the same age (a single cohort colony) that we could track through the first two weeks of life. Because we wished to repeatedly track the behaviours of all bees inside the colony, we video recorded micro-colonies of caged honey bees maintained in the lab, each micro-colony provided with a QMP lure to simulate queen presence (Fig. 1). Such pheromone presentation is highly effective, can maintain colony queen-right behaviours, and is used by beekeepers to create small nuclei^28^,^29^. We measured the most commonly exhibited behaviours, including attraction to QMP, as a measure of worker interaction with the queen.

## Results

We used bees from eight different colonies to conduct eight paired-treatment trials consisting of individually labeled 1-day old adult bees monitored for two weeks (364 bees in total). Bees were fed sucrose solution with the equivalent dose of 30,000 *N. ceranae* spores/bee (*Nosema* group) or sterile sucrose solution (control group). We measured day of death, midgut spore count at the end of the trial, and behaviours (Table 1) throughout the trial.

### No difference in survival between *Nosema* and control groups

Although there was slightly higher mortality in the *Nosema* exposed group (11% died by the end of 15 days as compared to 6% for the control group), mortality was not significantly different between these groups (treatment factor: L-R *χ*^2^_1_ = 0.26, *P* = 0.61). There was no effect of spore count upon adult death (L-R *χ*^2^_1_ = 0.46, *P* = 0.50), no significant interaction of treatment*spore count (L-R *χ*^2^_1_ = 0.26, *P* = 0.61), and no colony effect (L-R *χ*^2^_7_ = 12.48, *P* = 0.09).

### *Nosema* treatment altered bee behaviours

On average, *Nosema*-exposed bees were 21,935-fold more infected (Kruskal-Wallis test, *χ*_1_^2^ = 256.2, *P* < 0.0001) than control bees. Only 1.5% of control bees had spores, and these were minimally infected (Fig. 2).

Multiple behaviours changed over time in both treatment groups. Detailed statistics are presented in Table 2. There was a significant effect of day for trophallaxis, antennating, allo-grooming, auto-grooming, grooming dancing, shaking signaling, cell inspecting, pollen visiting, and standing (*P* ≤ 0.004). In general, these behaviours tended to decrease over time, with the exception of walking, which increased over time.

Bees in the *Nosema* treatment group exhibited a significantly higher level of food exchange (trophallaxis, *P* = 0.006) than bees in the control group (Fig. 3a). There was no significant interaction of treatment*day (*P* = 0.037, *NS*_*SB*_). Over the entire trial, *Nosema*-treated bees exhibited 1.4-fold higher levels of trophallaxis than control bees. Treatment was not significant for any other scored behaviour (*F*_1, 333_ ≤ 2.714, *P* ≥ 0.100, Table 2). *Nosema* treatment increased average trophallaxis per bee (ANOVA_non-RM_: *F*_1,332_ = 7.23, *P* = 0.008).

There was a significant treatment*day interaction for walking (*P* < 0.001) and QMP attraction (*P* = 0.015). During the last two days of the trial (Fig. 3b), walking significantly increased by 1.4-fold (contrast test: *F*_1, 1790_ = 43.45, *P* < 0.001). *Nosema* treatment increased average walking per bee (ANOVA_non-RM_: ***F***_**1,**332_ = 14.96, *P* = 0.0001).

QMP attraction significantly decreased by 0.7-fold (contrast test: *F*_1, 618_ = 6.41, *P* = 0.011, Fig. 3c) in the *Nosema*-treated bees relative to the control bees. *Nosema* treatment increased average queen attraction per bee (ANOVA_non-RM_: *F*_1,332_ = 5.94, *P* = 0.015).

### Relatively uninfected bees within the *Nosema* treatment behaved differently

Bees were group-fed an average dose of 30,000 spores at the start of the trial. Thus, bees with 30,000 spores or less were either uninfected and had retained the fed spores in their midguts or were very weakly infected. We therefore call these bees “relatively uninfected”. Within the *Nosema* treatment group, 22% of bees were relatively uninfected. Bees with more than 30,000 spores at the end of the trial were considered highly infected. We collected video data on 181 bees in the *Nosema* treatment. Of these bees, 141 were highly infected (6,282,252 ± 6,684,402 spores/bee) and 40 were relatively uninfected (7,625 ± 10,252 spores/bee) upon adult death.

There was a significant effect of day upon trophallaxis, walking, auto-grooming, grooming dancing, shaking, signaling, cell inspecting, pollen visiting, and standing (*P* ≤ 0.021). Detailed statistical results are in Table 3. For these behaviours, there was a tendency for the behavioural rates to decrease between days 2 to 14, except for walking, which increased.

Relatively uninfected bees exchanged food less often with nest mates (trophallaxis, *P* = 0.001) as compared to infected bees. The relatively uninfected bees also walked less (*P* < 0.001) and had higher QMP attraction (*P* = 0.014). Upon closer inspection, the relatively uninfected bees exhibited decreased trophallaxis during the first week of the trial (contrast test: days 2–8, *F*_1, 350_ = 8.220, *P* = 0.004, Fig. 4a). Towards the end of the trial, these relatively uninfected bees also walked less (contrast test: days 8–14, *F*_1, 125_ = 15.000, *P* < 0.001, Fig. 4b) and had higher QMP attraction (contrast test: days 13–14, *F*_1, 749_ = 6.720, *P* = 0.009, Fig. 4c) than highly infected bees.

We found significant interactions (Table 3) between infection level and day for visiting pollen (*P* = 0.021) and standing (*P* = 0.004), but there were no consistent overall trends and no significant contrast differences over any contiguous set of days for these behaviours (*P* > 0.025).

## Discussion

*Nosema ceranae* is a major disease that also accelerates honey bee age polyethism. We provide detailed data showing that young worker behavioural development accelerates as a result of *N. ceranae* infection. We found evidence for two accelerated behaviours: 12 days after exposure, walking significantly increased and attraction to Queen Mandibular Pheromone (QMP) decreased as compared to control bees. This change in QMP attraction could limit queen exposure to infection, which typically reaches a peak 10–12 days after first exposure^30^. Bees in the *Nosema*-treated group also exhibited significantly higher rates of trophallaxis than control bees, perhaps reflecting increased hunger and food begging^16^,^31^. Within the *Nosema*-treated bees, highly infected bees (>30,000 spores/bee) exhibited the same behavioural changes: increased walking and decreased QMP attraction towards the end of the trial and higher overall rates of trophallaxis.

Workers in our micro-colonies exhibited other bee behaviours, including communication signals such as the grooming dance, which elicits grooming from receivers^32^, and the shaking signal, which helps to reallocate colony labour^33^. Production of these signals significantly decreased over time (Table 2), but was not affected by *Nosema* treatment. Similar results were observed in a study by Natsopoulou *et al.*^21^, who observed bees beginning at four days of adult age and found that they retained a wide behavioural repertoire under parasitic stress.

McDonnell *et al.*^34^ used observation colonies but did not find behavioural differences between *Nosema*-infected and healthy bees. However, this study observed randomly selected bees within each treatment group for 15 min per trial. In contrast, our results show that longer-term observations (144 min per trial equally spaced over 12 days) are evidently needed to reveal *Nosema* effects on age polyethism, as also demonstrated by Natsopoulou *et al.*^21^.

Because our goal was to conduct detailed, long-term observations of behavioural changes, we used single-cohort micro-colonies so that we could repeatedly measure behaviours over time. While laboratory assays rarely offer a complete representation of behaviours in the natural environment, they allow for manipulations and repeatability seldom matched by field conditions. A primary advantage of our approach is that it allowed us to observe all bees within a micro-colony over an extended period. A disadvantage is that natural colonies are normally much larger and have bees of different ages performing different tasks. However, the single-cohort colony approach has proven valuable for understanding age polyethism in honey bees^35^, and using micro-colonies facilitated more thorough behavioral observations. Although it is possible to track a large number of bee movements over time in a largely automated way^36^, this technology, to date, does not automatically measure most of the detailed behaviours that we commonly observed.

Previous studies have shown that micro-colonies can be used to determine the effects of pathogens on honey bee health and these results can correspond to results with full-size colonies^37^. Our approach was sufficient to show decreases in queen attraction, matching the preliminary results of a study using *Nosema*-infected bees studied in full colonies^38^. Moreover, recent research^21^,^39^ demonstrated that workers infected with *Nosema* significantly decreased their attraction towards a living honey bee queen.

In our full experiment, the micro-colonies did not contain brood, and thus we could not monitor nursing behaviours that would be typical of very young bees. However, we conducted a preliminary trial with more realistic conditions: micro-colonies containing brood and adults of different ages (Supplemental Information, SI). We changed our procedures for the full experiment because of issues with this design, mainly poor brood survival, but the data showed the same trends: bees fed *Nosema* significantly increased trophallaxis and walking (Fig. S1). In this preliminary trial, QMP attraction was not videotaped, although chance observations of such attraction led us to reposition the QMP lure and monitoring attraction in our full experiment.

In a typical honey bee colony, workers have some behavioural flexibility and can perform multiple tasks at any given age^17^. However, the first 2 to 3 weeks are generally spent tending the queen and the brood and maintaining the nest^17^. Queen tending then decreases as workers age^40^. The endocrine factor, juvenile hormone^41^ increases as workers age^13^,^42^ and is a primary regulator of these behavioural changes^42^. Goblirsch *et al.*^13^ showed that young workers infected with *Nosema sp.* had higher JH titres than uninfected bees and initiated premature foraging. McQuillan *et al.*^42^ demonstrated that young workers treated with JH became less attracted to a QMP lure. We observed the same behaviour in our *Nosema*-infected bees. This decrease in QMP attraction may have resulted from an increased level of JH. Attraction to the QMP lure, a proxy for queen-tending, decreased by 0.7-fold (*Nosema* vs. control group) and 0.6-fold (within the *Nosema* group) in infected bees during the last two days of the trial. Similarly, Wang and Moeller^38^ reported a 0.2-fold decrease in queen attendance in 6–10 day old bees infected with *Nosema* in observation colonies.

We measured 1.4-fold increases in walking from bees that were 8–14 days old (Figs 3b and 4b). This observation is also consistent with accelerated age polyethism. Johnson^40^ showed that middle-aged bees (14–18 days old) naturally performed 6-fold more walking than nurse bees (4–8 days old). Increases in walking also correspond to a general increase in activity reported in *Nosema*-infected bees^21^,^38^. Accelerated age polyethism can also be the result of other types of demographic stressors, leading to positive feedbacks that could drive the rapid depopulation of a colony^43^.

Trophallaxis was significantly higher in *Nosema*-group bees as compared to control bees throughout the 14-day trial and was also higher in infected as compared to relatively infected bees within the *Nosema* group during the first 8 days of the trial. Interestingly, this difference was evident almost immediately, beginning at day 2 (equivalent to 24 hours post-exposure after the consumption of the full 30,000 spore dose). What accounts for this rapid effect? Most studies on the effect of *N. ceranae* on bee physiology or behaviour tend to begin data collection at least one week post-exposure^13^,^22^,^31^,^44^,^45^. Although mature *N. ceranae* spores are not detectable until 4 days post-infection, the proliferating vegetative stage is already quite active^30^. In cell culture, this vegetative stage begins 16 hours after inoculation and the first spores can be detected after 48 hours^46^. Thus, the early infection stage could have caused the increase in trophallaxis that we observed.

These changes in behaviour could serve as host defense mechanisms, result from parasite manipulation, or both^47^. With respect to host defense, diseased individuals that accelerate their age polyethism will more rapidly become foragers^38^. In consequence, they will spend more time outside the nest and potentially reduce disease transmission within the nest^48^. For example, immune-suppressed honey bees avoid social contact with healthy nest mates^26^. Reduced contact with the queen could also help limit the spread of infection, given that the queen is a nexus of colony contacts. Alternatively, *Nosema* may be manipulating its host to increase transmission^49^. Infected bees prefer the higher temperatures of the inner hive, benefiting pathogen transmission^22^. Increased trophallaxis (Figs 3a and 4a) could increase the spread of infection, particularly if spores are orally transmitted^14^. The actual roles of these behaviours in benefiting or harming the colony are unclear. Future studies testing the efficacy of self-removal for reducing infection spread and the extent of *Nosema* transmission through trophallaxis would be beneficial. However, our results add to growing evidence that *N. ceranae* infection prematurely accelerates honey bee age polyethism, thereby perturbing a delicate balance that is important to the correct allocation of colony tasks.

## Methods

### Video observation cages

**Figure 1:**
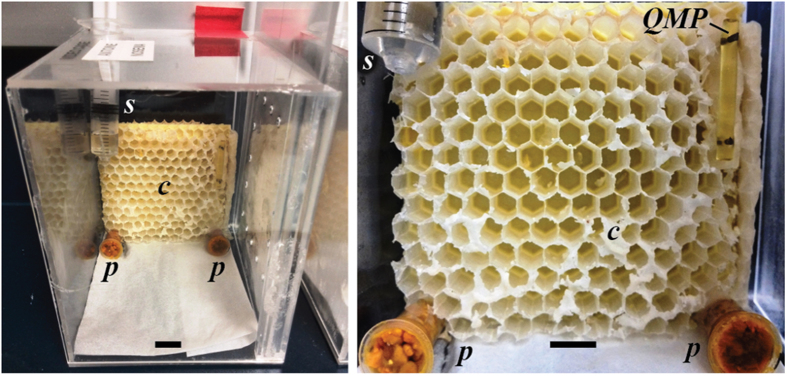
Detail of a video observation cage showing the comb = *c*, pollen = *p*, sucrose solution = *s*, and Queen Mandibular Pheromone = *QMP*. The horizontal scale bars indicate 1 cm. The video observation window has been removed in these photos for a clearer image. Combs originally contained honey stores and some brood, but were carefully cleaned and sterilized before use (see Methods).

The experimental cages were custom made (12 × 8 × 12 cm) of transparent acrylic with a sliding plastic door pierced with holes for ventilation and a hole in the top through which we inserted a 5 ml syringe containing sucrose solution to feed the bees (Fig. 1). Inside, we placed an empty, sterile honey bee comb with its back against the cage so that bees could only access the side facing the video camera. The camera viewing window was removable so that it could be swapped out for a clean window. Each cage was provided with *ad-libitum* 50% (1.8 M) sterile sucrose solution and two 1.5 ml centrifuge tubes, each with 1 g of a pollen mixture: 90% (w/w) irradiated corbicular pollen (Mann Lake, Hackensack, Minnesota, USA) mixed with tap water^50^. Each cage was also fitted with a half strip of a queen mandibular pheromone (QMP) lure (Bee Boost, PheroTech Inc., Delta, Canada) to simulate the presence of a queen. All cages with bees were kept inside a dark 33 °C and 70% humidity incubator. To ensure initially sterile conditions, we soaked all combs, cages, feeding syringes, and pollen tubes in a 10% bleach solution for at least 30 min, followed by soaking in and multiple rinses with deionized water. All items were then sterilized for 1 hr with ultraviolet light inside an AirClean 600 sterile laminar flow hood (www.BioExpress.com) and then dried inside this hood for at least 24 hrs.

### *Nosema* spore preparation

*Nosema ceranae* spores were originally obtained from infected *A. cerana* workers in Thailand and fed to *A. mellifera* workers in La Jolla, California. Spore-producing bees were not fed pollen, only pure 55% (w/w) sucrose solution to ensure that gut contents consisted mainly of spores. To obtain spores, we dissected out adult honey bee midguts, homogenized them in sterile double distilled water (ddH_2_0), and vacuum-filtered them through Fisherbrand P8 filter paper (Fisher Scientific, Pittsburgh, Pennsylvania, USA) with 20–25 μm pores. We collected the filtrate in microcentrifuge tubes that we centrifuged (Eppendorf 5415D centrifuge, Hauppauge, New York, USA) at 9279 g (Relative Centrifugal Force) for 15 min^51^,^52^. We then removed the supernatant and re-suspended the pellet in sterile ddH_2_0 water. This procedure resulted in fairly pure spore preparations as determined with a microscope. We measured spore concentrations with a haemocytometer in a compound microscope^53^,^54^. We infected new bees and extracted fresh spores for each trial.

We sequenced the DNA of our *Nosema* spores to determine its species identity. To obtain the DNA, we froze spore stock with liquid nitrogen and crushed the spores with a pestle before DNA extraction with a Bioneer Accuprep Genomic DNA extraction kit. We used primer pairs NoscRNAPol-F2 (AGCAAGAGACGTTTCTGGTACCTCA) and NoscRNAPol-R2 (CCTTCACGACCACCCATGGCA)^55^. The resulting spore DNA was then amplified with PCR and sequenced using standard methods^55^. Cross-checking with Genbank sequences confirmed that we were infecting our bees with *N. ceranae*.

### Treatment of bees

**Figure 2:**
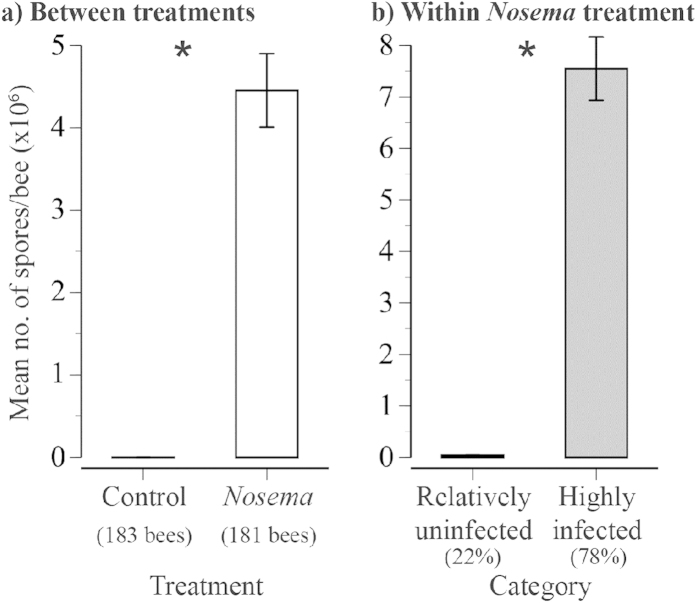
Effect of *Nosema* treatment upon the mean number of midgut spores upon adult death (**a**) between treatments (sample size shown) and (**b**) within the *Nosema* treatment (% within each category shown). Asterisks indicate significant differences.

These studies were conducted at UCSD, La Jolla, California. USA. We used nine colonies, a different colony for our preliminary trial (*N* = 108 bees, see SI) and each of our eight full experiment trials (*N* = 400 bees). New package colonies were obtained in the spring and treated with fumagilin dicyclohexylammonium (Fumagilin-B) in 25 mg/l of 2.0 sucrose solution (3.8 l/colony). After this initial treatment, we did not use the colonies for 60 days to allow the antibiotic to dissipate (method of Milbrath *et al.*^56^, detailed information on generating *Nosema*-free colonies from author Z. Huang). Huang *et al.*^57^ analyzed fumagillin residues and calculated that *N. ceranae* could hyperproliferate 2 to 5.5 months after treatment cessation. A 60-day waiting period yielded uninfected bees that could become highly infected with *N. ceranae* in our study (Fig. 2) and in Milbrath *et al.*^56^.

For each trial, a frame of *Nosema*-free capped brood from one of the research colonies was collected 1–2 days before brood emergence and incubated in the lab at 33 °C and 70% humidity. We randomly selected 50 bees upon emergence, individually numbered them with bee tags attached with cyanoacrylate adhesive (Queen Marking Kit, www.beeworks.com) and divided them into two cages, a control and a *Nosema* test cage (25 bees per cage). Each trial consisted of one control and one *Nosema* cage running in parallel. The control treatment bees were only fed sterile 50% sucrose solution. The *Nosema* treatment bees were group fed 750,000 *Nosema* spores in a 1 ml solution of 50% sucrose, equivalent to 30,000 spores per bee. On average, the bees consumed these 30,000 spores in 4.1 ± 1.2 days and were fed sterile sucrose solution, like the control cage, for the remainder of the experiment.

### Behavioural analysis

Each trial covered the first 15 days of adult bee life. Every 4 hrs (00:00, 04:00, 08:00, 12:00, 16:00, and 20:00), we video-recorded (H.264 Network DVR, www.cibsecurity.com) the bees for 30 min. For each cage, we used a CCD camera (Sony 1/3 Varifocal Camera, 700 TVL, 976 × 494 pixels) with infra-red LED lights (850 nm) that are invisible to bees^58^ to illuminate the bees in the dark incubator. To eliminate LED reflections, we placed each camera directly against the camera-viewing window of the cage. Subsequently the behaviours were scored (Table 1) by manual inspections of the videos during the first and the last minute of each 30 min recording period and summed to create behaviour counts for that 30 min period. Each bee could perform multiple behaviours within each 1-min observation interval. Behaviours typically lasted a few seconds and thus performing one behaviour did not prevent the bee from performing other behaviours. We focused on the 13 most common behaviours that we observed. Video data transcribers were extensively trained as a group to record the behaviours, and their work was verified before they were allowed to collect data on their own.

### Inclusion of data for analyses

We excluded three bees from the behavioural analysis of control trials (1.5% of control bees) because they had *Nosema* spores. These bees were minimally infected compared to *Nosema* group bees (Fig. 2), and their infection levels (2.7 ± 1.2 spores per bee haemocytometer count) may have arisen from subsequent contamination. In total, we analyzed the behaviours of 183 bees in the control group and 181 bees in the *Nosema* group (78% of *Nosema* group bees had >30,000 spores and 22% had ≤30,000 spores per bee, the average dose fed to these bees).

### Statistical analyses

We log-transformed all behavioural measures and used Repeated-Measures Analysis of Variance (ANOVA), REML algorithm (JMP Pro v11.2.0 software), with bee identity as a repeated measure and treatment (control or *Nosema*), day and the interaction treatment*day as fixed effects. In addition, each trial consisted of two cages (one control and one *Nosema* cage). Cage identity was therefore incorporated in our models through the inclusion of colony and treatment. We initially tested time of day in our models, but excluded this variable from our final models^59^ because it was not significant for any behaviors (*P* ≥ 0.22).

For behaviours that showed changes over time, we applied post-hoc contrast tests to test for treatment differences. In our initial models, we included observer as a random factor, but found that it accounted for <1% of model variances. This indicates that observer bias was fairly low, and we therefore did not include observer as a factor in our final models.

Measurement of the *Nosema* spore counts in the bees exposed to *N. ceranae* spores, revealed different levels at the end of the experiment in spite of all bees sharing the same sugar solution with the same concentration of *Nosema* spores. To determine if this variable level of infection corresponded to bee behaviours, we divided our data into two subgroups: bees that showed elevated spore levels upon death, a sign of active infection, and a second group, for those bees that retained less than 30,000 spores, a sign of being uninfected or less infected, ran the same model, and applied contrast tests as appropriate. In these models, incorporating colony as a random effect included the effects of trial and cage because each trial was conducted with a different colony and consisted of one cage of bees.

Because we analyzed the data twice (control vs. *Nosema* treatment and infected vs. uninfected within the *Nosema* treatment, we applied a sequential Bonferroni correction^60^ with *k* = 2, yielding an adjusted alpha value of 0.0253, to correct for potential type I statistical error. Tests were only considered significant if *P* < 0.0253. Tests that fail this corrected alpha are denoted as *NS*_*SB*_. To explore the effects of *Nosema* infection in detail, we measured 13 different behavioural responses (Table 1) to our treatment. However, we had no *a priori* expectations of how these behaviours would change. Moreover, a Bonferroni *k* that is the sum of all these behavioural measures would not be appropriate because the Bonferroni correction is too conservative for large *k*, is susceptible to type II statistical errors, and inhibits detailed analyses of the phenomena being studied^60^,^61^. We therefore followed the procedures suggested by Moran^60^ in presenting our analyses and applied the *k* = 2 Bonferroni correction as a compromise between the problems of type I and type II statistical error.

**Figure 3:**
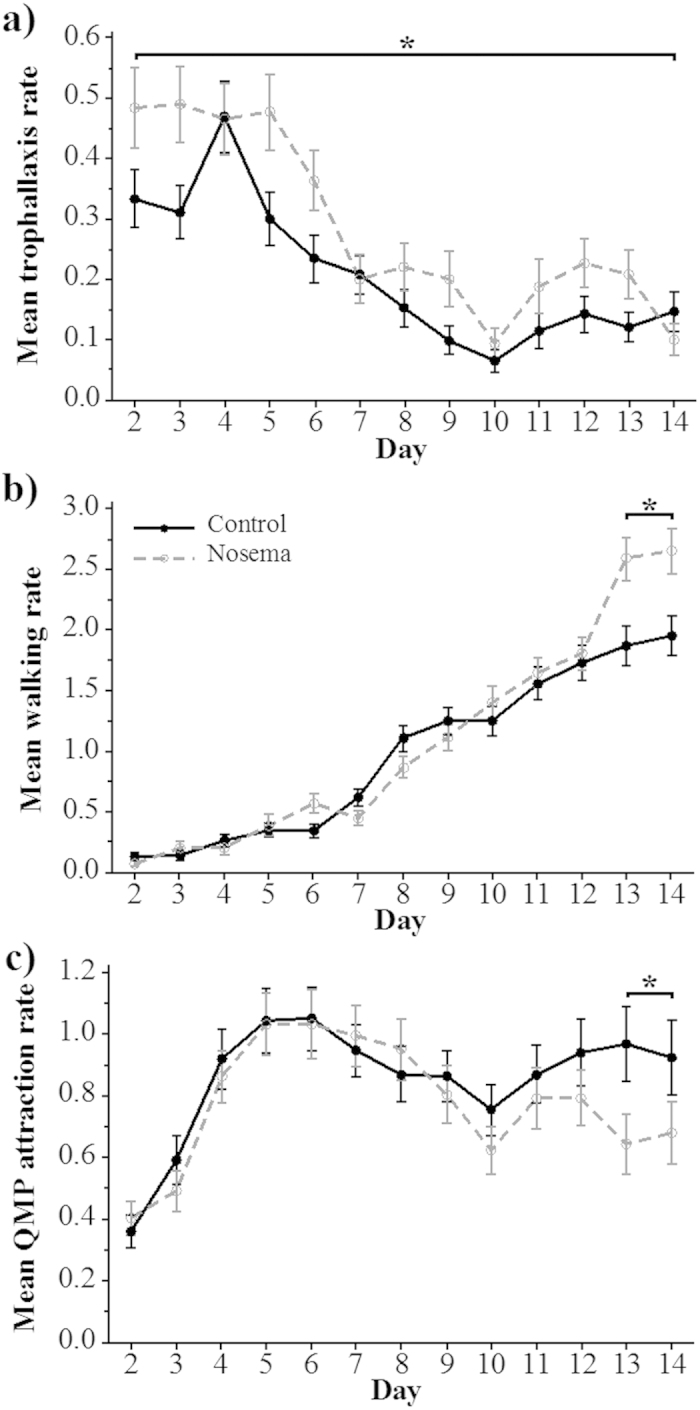
Comparisons between the behaviours of bees in the control and *Nosema* treatments. Mean daily rates (standard error bars shown) for (**a**) trophallaxis, (**b**) walking and (**c**) QMP attraction behaviours in control and *Nosema*-treated groups of honey bees. Significant contrast test differences are indicated with an asterisk.

**Figure 4:**
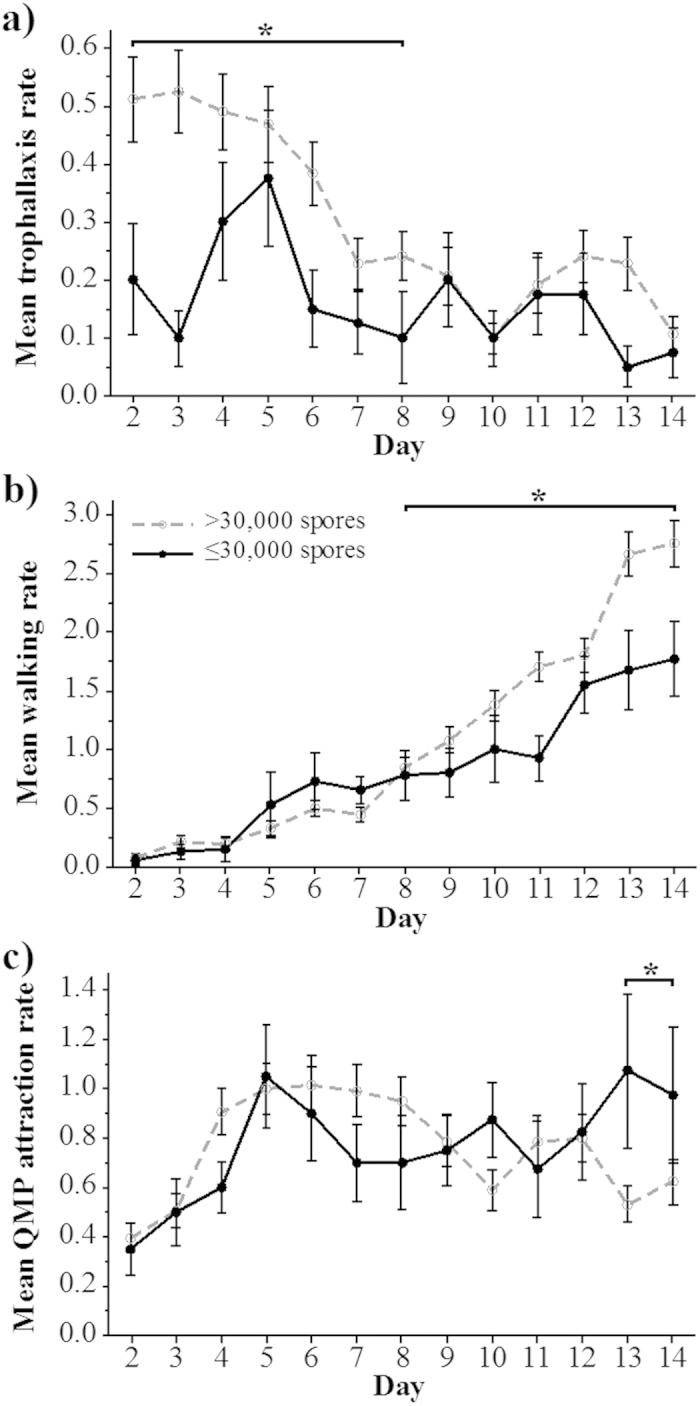
Comparisons between uninfected bees and infected bees in the *Nosema* treatment. Mean daily rates (standard error bars shown) for (**a**) trophallaxis, (**b**) walking and (**c**) QMP attraction behaviours in relatively uninfected bees with ≤30,000 spores and infected bees with >30,000 spores. An asterisk indicates significant contrast test differences.

A repeated-measures analysis is suited to the nature of our experimental design, but as an alternative (but not independent) analysis, we simply averaged the behaviors of each bee and compared the effects of treatment upon three behaviors over relevant time spans (see Figs 3 and 4): trophallaxis (days 2–8), walking (days 13–14), and queen attendance (days 13–14). For these analyses, we used ANOVA with treatment (control vs. *Nosema*) as a fixed effect and colony as a random effect. We denote these analyses as ANOVA_non-RM_.

We tested survival using a proportional hazards fit model, including colony and spore count upon adult death as a factor. The data were right-censored because all trials terminated when bees were 15 days old. We report our results^25^ as mean ± 1 standard deviation.

## Additional Information

**How to cite this article**: Lecocq, A. *et al.* Parasite infection accelerates age polyethism in young honey bees. *Sci. Rep.* **6**, 22042; doi: 10.1038/srep22042 (2016).

## Supplementary Material

Supplementary Information

Supplementary Dataset 1

Supplementary Dataset 2

Supplementary Dataset 3

## Figures and Tables

**Table 1:** List of observed and recorded behaviours and their definitions.

Behaviour	Description
Trophallaxis	Nest mate exchange of food. The receiver extends its proboscis into the donor’s mouthparts; the donor opens its mouthparts, and regurgitates food.
Walking	A bee walking around on the comb
QMP attraction	A bee interacting with the QMP lure by contact with antennae or tongue
Antennating	Antennal contact, head to head between two bees, with no food exchange
Allo-grooming	A bee running a nest mate’s body parts through its mandibles
Auto-grooming	A bee running its own body parts through its mandibles
Grooming dance	A bee stands and vibrates her whole body dorso-laterally. Sometimes body vibration is mixed with brief bouts of self-grooming
Shaking signal	Nest mate vigorously and rhythmically shaking her body dorso-ventrally while gripping another bee or the comb^33^
Fanning	Flapping wings while standing in hive
Cell inspecting	Momentary insertion of the anterior portion of the head into an empty cell
Visiting sugar	A bee imbibing the sugar solution
Visiting pollen	A bee consuming pollen
Standing	A bee standing stationary on the comb

These were the most 13 commonly observed behaviours. References for these definitions are provided, as appropriate.

**Table 2:** Statistical results of control versus *Nosema*-treated bees for all the recorded behaviours.

Behaviour	Model Fit (R^2^)	Treatment		Day		Treatment*Day		Colony Effect (%)
		F _1, 333_	P-value	F _1, 4102_	P-value	F _1, 4102_	P-value	
Trophallaxis	0.161	7.701	**0.006**	190.896	**<0.001 (−)**	4.362	**0.037**	8.2
Walking	0.491	1.219	0.270	2041.778	**<0.001 (+)**	29.001	**<0.001**	17.7
QMP attraction	0.262	1.039	0.309	3.681	0.055	5.902	**0.015**	4.9
Antennating	0.128	0.780	0.378	28.872	**<0.001 (−)**	0.114	0.736	7.0
Allo-grooming	0.079	2.413	0.121	14.619	**<0.001 (−)**	0.001	0.978	3.7
Auto-grooming	0.305	<0.001	0.987	189.574	**<0.001 (−)**	2.607	0.106	16.6
Grooming dance	0.042	0.681	0.410	11.728	**<0.001 (−)**	2.622	0.105	0.9
Shaking signal	0.167	0.005	0.941	8.068	**0.004 (−)**	0.430	0.512	1.3
Fanning	0.078	0.170	0.681	1.815	0.178	0.692	0.405	1.2
Cell inspecting	0.268	2.714	0.100	48.800	**<0.001 (−)**	1.413	0.235	17.3
Visiting sugar	0.052	0.649	0.421	0.698	0.404	1.654	0.199	1.7
Visiting pollen	0.115	0.893	0.345	189.385	**<0.001 (−)**	0.193	0.660	5.1
Standing	0.268	0.005	0.945	51.013	**<0.001 (−)**	1.314	0.252	5.5

Plus/minus signs indicate the direction of the changes from day 2 through to 14.

**Table 3:** Statistical results of comparisons between bees with ≤30,000 *Nosema* spores and those with >30,000 spores for all the recorded behaviours.

Behaviour	Model Fit (R^2^)	Infection Level		Day		Infection Level*Day		Colony Effect (%)
		F _1, 176_	P-value	F _1, 2170_	P-value	F _1, 2170_	P-value	
Trophallaxis	0.172	1.697	0.194	39.339	**<0.001 (−)**	10.577	**0.001**	7.3
Walking	0.479	2.335	0.128	699.919	**<0.001 (+)**	26.783	**<0.001**	13.8
QMP attraction	0.263	0.477	0.491	2.320	0.128	5.984	**0.014**	6.2
Antennating	0.159	0.117	0.733	11.331	**<0.001 (−)**	0.710	0.400	9.1
Allo-grooming	0.097	1.493	0.223	2.145	0.143	0.117	0.732	8.5
Auto-grooming	0.306	0.880	0.349	101.576	**<0.001 (−)**	1.000	0.317	17.9
Grooming dance	0.040	0.081	0.776	5.305	**0.021 (−)**	0.645	0.422	1.3
Shaking signal	0.078	0.059	0.808	7.340	**0.007 (−)**	0.595	0.440	1.3
Fanning	0.075	0.501	0.480	0.228	0.633	2.704	0.100	1.2
Cell inspecting	0.306	0.000	0.990	15.296	**<0.001 (−)**	2.899	0.089	20.0
Visiting sugar	0.046	2.616	0.108	0.064	0.800	0.282	0.595	2.0
Visiting pollen	0.112	0.657	0.419	39.341	**<0.001 (−)**	5.354	**0.021**	6.4
Standing	0.263	0.310	0.578	29.556	**<0.001 (−)**	8.289	**0.004**	5.4

Plus/minus signs indicate the direction of the changes from day 2 through to 14.
