# Supplementary material for: Parasite infection accelerates age polyethism in young honey bees
Source: Sci Rep. 2016 Feb 25;6:22042. doi: 10.1038/srep22042 (PMC4766577; doi:10.1038/srep22042)
Supplement: Supplementary Information [file srep22042-s1.pdf]

Parasite infection accelerates age polyethism in young honey bees

Antoine Lecocq[1,2,*], Annette Bruun Jensen[2], Per Kryger[3], and James C. Nieh[1]

[1] Division of Biological Sciences, Section of Ecology, Behavior, and Evolution, University of California San Diego, 9500 Gilman Drive, MC 0116, La Jolla, California, 92093–0166, United States of America

[2] University of Copenhagen, Department of Plants and Environmental Sciences - PLEN, Thorvaldsensvej 40, 1871 Frederiksberg C, DK

[3] Aarhus University, Department of Agroecology - Entomology and Plant Pathology, Forsøgsvej 1, 4200 Slagelse, DK

* Corresponding author

antoine2@outlook.com

Supplemental Information

Supplemental Methods

In a preliminary experiment, we conducted a single trial with 108 bees (54 bees per cage, one cage for the *Nosema* treatment and one for the control treatment). Unlike subsequent trials, the combs used for these cages contained an approximately equal proportion of stored honey and brood, thus more closely approximating a colony. In addition, adult bees were not the same age, but rather were bees that were on the comb sections before they were cut out. They therefore contained bees of multiple ages that typically aggregate on the brood comb. These bees were initially placed into a common cage, removed for labeling, and randomly assigned to the control or *Nosema* cages.

We scored the same set of behaviors, except for QMP attraction. As in the other trials, we placed a QMP lure inside each cage, but attached to the cage door, not to the comb and thus not in view of the video camera. This preliminary trial therefore did not include an analysis of attraction to the QMP. Because we sometimes observed bees contacting the QMP lure, we decided to position the lure on the comb and score this behaviour in the subsequent full experiment. Behaviour was otherwise analyzed as described for the full experiment. We scored behaviors for 2 min each 4 hrs, but did so every two days. In the full experiment, we scored behaviors every day. We made other changes in the full experiment because (1) the brood did not survive to the end of the trial, (2) we were concerned about the potential for spores present in the comb wax, and (3) using adult bees of unknown ages is problematic. In the full experiment, we therefore used sterilized combs without brood or stored honey and bees that were all the same age.

These data were analyzed in the same way as the full data (see paper). A Bonferroni correction was not applied to analyses of this preliminary trial since this data was only analyzed once.

Results & Discussion

In this preliminary trial (Fig. S1a), as in the full experiment (see paper), trophallaxis was elevated in *Nosema*-treated as compared to control bees (treatment effect: $F_{1, 78}$=9.78, P=0.003). This increase in trophallaxis in the *Nosema*-treated bees matches the results of the full experiment (Fig. 3a). Likewise, there was an effect of time ($F_{1, 78}$=5.11, P= 0.027). The interaction treatment*time was not significant ($F_{1, 78}$=3.06, P=0.08). Unlike the full experiment, trophallaxis in *Nosema*-treated bees increased markedly at day 14, perhaps due to methodological differences. However, the overall trend of increased trophallaxis was the same.

In the preliminary trial (Fig. S1b), walking was also elevated in *Nosema*-treated as compared to control bees (treatment effect: $F_{1, 78}$=4.06, P=0.04). Time and the interaction treatment*time were not significant ($F_{1, 78}\leq$0.72, $P\geq$0.40). In the full experiment, the walking increased towards the end of the 14-day trial, whereas the preliminary data showed a roughly consistent elevation over time. These differences may have arisen from the methodological differences, but the overall trend of increased walking was similar.

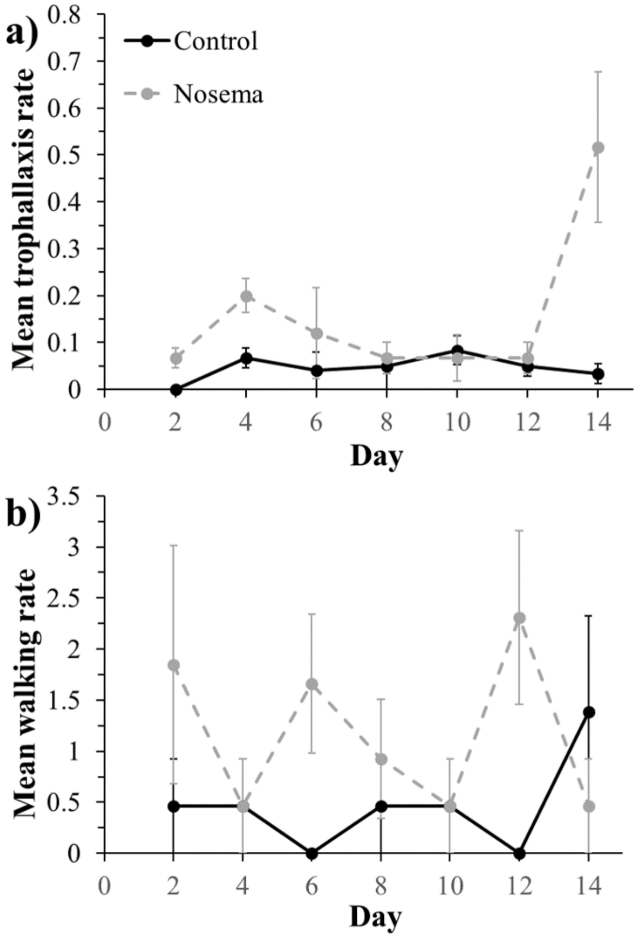

Figure S1. Effect of *Nosema* treatment in the preliminary experiment upon (a) the number of trophallaxis and (b) walking events per bee. Standard error bars are shown.

Supplementary Data S1

Trial: Which trial these bees were part of

Treatment: Either control group or Nosema group

Colony: Name of the colony used for a particular trial (different colony for each trial)

BeeID: Unique identifying name for each bee

ID: Original tag number of the bee in the group

Spore count: Amount of spores found in the bee by microscopic analysis

Day of Death: Day at which the bee died (if it died prior to the end of the trial)

Alive at end of trial? (censoring): 1= bee was still alive at the end; 2= bee died prior to the end

Binary infection level: Categorical variable linked to "Spore count"; ≤30K= Bee with equal or less than 30,000 spores at the end of the trial; >30K= Bee with more than 30,000 spores at the end of the trial

Supplementary Data S2 and S3

Treatment: Either control group or Nosema group

Trial: Which trial these bees were part of

Colony: Name of the colony used for a particular trial (different colony for each trial)

Cage: Specific i.d for each cage used in the trial. Combination of trial number and treatment

Observer: The name of the scientist responsible for the data analysis and behaviour recording

Date: Date during which the behaviours took place, for each trial

Day: Numerical value of the date, days run from 2 to 14

Period: Aggregated days into 3 periods linked to the development times of a *Nosema* sp. Infection; Period 1= days 2 to 5; Period 2= days 6 to 10; Period 3= days 11 to 14

ID: Original tag number of the bee in the group

BeeID: Unique identifying name for each bee

Spore count: Amount of spores found in the bee by microscopic analysis

Ordinal infection level: Categorical variable linked to “Spore count”; no spores= 0 spores; ≤30K= Bee with equal or less than 30,000 spores at the end of the trial; >30K= Bee with more than 30,000 spores at the end of the trial

Binary infection level: Categorical variable linked to “Spore count”; ≤30K= Bee with equal or less than 30,000 spores at the end of the trial; >30K= Bee with more than 30,000 spores at the end of the trial

Spores?: Did with see spores in the bee? No spores= no; spores= yes

Standing; Walking; Trophallaxis; Antennating; Grooming self; Allo-grooming; Groom dance; Inspect cell; Fanning; Shake sign; Sugar visit; Pollen visit; Queen Attendance: Please refer to table 1 for the descriptions of the observed behaviours. Behavioural rates are per day.

LogStanding; LogWalking; LogTrophallaxis; LogAntennating; LogGrooming self; LogAllo-grooming; LogGroom dance; LogInspect cell; LogFanning; LogShake sign; LogSugar visit; LogPollen visit; LogQueen Attendance: Log transformed data used for analysis and modelling.
